# Evaluation of Plasma Nitric Oxide and Serum Endothelial Nitric Oxide Synthase in Pulmonary Hypertensive Dogs: A Clinical and Echocardiography Investigation

**DOI:** 10.3390/vetsci12050486

**Published:** 2025-05-16

**Authors:** Siwayu Rattanakanokchai, Numfa Fungbun, Ketmanee Senaphan, Supranee Jitpean, Trasida Ployngam

**Affiliations:** 1 Veterinary Teaching Hospital, Faculty of Veterinary Medicine, Khon Kaen University, Khon Kaen 40002, Thailand; siwara@kku.ac.th; 2 Division of Companion Animal Medicine, Faculty of Veterinary Medicine, Khon Kaen University, Khon Kaen 40002, Thailand; numfa@kku.ac.th; 3 Division of Physiology, Faculty of Veterinary Medicine, Khon Kaen University, Khon Kaen 40002, Thailand; ketmse@kku.ac.th; 4 Division of Surgery, Faculty of Veterinary Medicine, Khon Kaen University, Khon Kaen 40002, Thailand; supraneeji@kku.ac.th

**Keywords:** biomarkers, dogs, echocardiography, endothelial nitric oxide synthase, nitric oxide, pulmonary hypertension, right-sided congestive heart failure

## Abstract

Nitric oxide (NO), an endogenous vasodilator, has been proposed as a biomarker for pulmonary hypertension (PH) in humans. NO is synthesized by endothelial nitric oxide synthase (eNOS). Alterations in NO/eNOS have not been studied in dogs with PH. We assessed alterations in NO and eNOS in the blood of dogs with PH (*n* = 17) and healthy dogs (*n* = 10) and analyzed their correlations with echocardiographic parameters. The results showed significantly higher plasma NO and serum eNOS levels in dogs with PH compared with healthy dogs. Dogs with PH and ascites (*n* = 11) had significantly lower plasma NO levels than those without ascites (*n* = 6) and presented a decreasing eNOS trend. In dogs with PH, plasma NO was positively correlated with left ventricular hemodynamics, right ventricular compliance, and pulmonary distensibility, but was negatively correlated with pulmonary vascular resistance and right cardiac remodeling. Serum eNOS was positively correlated with the main pulmonary artery diameter. Increments in NO/eNOS reflected compensatory responses to cardiovascular changes in PH. These compensations were downward in the advanced stages. Other factors may also impact NO/eNOS compensation. Although the role of NO/eNOS as biomarkers for PH in dogs remains equivocal, they may indicate compensatory consequences of cardiovascular alterations.

## Simple Summary

Pulmonary hypertension (PH) is characterized by high pulmonary arterial pressure. Its clinical signs include collapse, weakness, and difficulty breathing. Severe PH can lead to the development of syncope and ascites associated with right-sided congestive heart failure. Nitric oxide (NO)/endothelial nitric oxide synthase (eNOS) are elemental factors in NO/cGMP signaling that regulate vascular smooth muscle tone. To date, there are no studies on NO/eNOS in dogs with PH. Thus, the present study aims to assess alterations in NO/eNOS in dogs with PH, and their correlations with cardiovascular modifications. The results show significant increases in NO/eNOS levels in the blood of dogs with PH compared with healthy dogs, suggesting compensatory responses to cardiovascular changes.

## 1. Introduction

The American College of Veterinary Internal Medicine (2020) published a consensus statement defining pulmonary hypertension (PH) in dogs as a mean pulmonary arterial pressure (PAP) greater than 25 mmHg at rest [1]. Idiopathic or primary abnormalities, such as left heart diseases, respiratory diseases, pulmonary emboli/thrombi/thromboemboli, parasitic diseases, multifactorial causes, and unclear mechanisms, can cause PH in dogs [1]. Clinical manifestations of PH include syncope, exercise intolerance, respiratory distress, and cardiogenic ascites [2]. Measurement of PAP by right-heart catheterization is the gold standard for PH diagnosis. However, in veterinary clinical practice, echocardiography is more suitable due to its non-invasiveness and repeatability [3]. In addition, echocardiography can clinically evaluate the primary cardiac causes, severity, and consequences of PH in terms of anatomic and functional cardiovascular alterations. Echocardiographic findings of PH in dogs are represented by tricuspid regurgitation velocity and structural changes, which can be categorized into three major sites [1]: (1) right ventricular (RV) abnormalities, including flattening of the interventricular septum, reduced left ventricular size, RV hypertrophy, RV dilation, and RV systolic dysfunction; (2) pulmonary artery (PA) abnormalities, such as PA distension, increased pulmonic regurgitation velocity greater than 2.5 m/s, decreased pulmonary artery compliance, and a reduced RV outflow Doppler acceleration time to ejection time (AT/ET); and (3) enlargement of the right atrium and caudal vena cava [4,5,6,7,8,9,10]. The pathophysiology of PH can be attributed to three primary disease etiologies [1]: (1) pulmonary overcirculation, (2) increased pulmonary vascular resistance, or (3) a combination of both.

Nitric oxide (NO), an endogenous vasodilator, is synthesized from L-arginine by nitric oxide synthases (NOSs) [11]. NOSs exist in three isoforms: neuronal NOS (nNOS or NOS-1), inducible NOS (iNOS or NOS-2), and endothelial NOS (eNOS or NOS-3) [12]. Each isoform contributes to NOS signaling, promoting diverse biological functions of NO. Herein, focusing on vascular homeostasis by NO/cGMP signaling, the synthesis of NO within the endothelial layer is initiated by eNOS. NO diffuses to adjacent vascular smooth muscle cells, where it activates signal transduction. NO binds to soluble guanylate cyclase (sGC), an enzyme that converts guanosine triphosphate (GTP) to cyclic guanosine monophosphate (cGMP) [13]. Activation of cGMP-dependent protein kinase causes a decrease in intracellular calcium, resulting in a reduction in vascular muscle tone. In turn, the transmembrane protein caveolin conducts NOS to an inactive state until cytoplasmic calcium and calmodulin reach sufficient levels. Caveolin dissociation and NOS activation are mediated by the binding of calcium-activated calmodulin [14,15]. Impaired NO/cGMP signaling triggers endothelial dysfunction, which can cause hypertension, atherosclerosis, and heart failure. Mechanisms that interfere with the NO/cGMP pathway include a reduction in NOS activity, downregulation of cGMP, and acceleration of cGMP degradation. NOS activity is impeded by asymmetric dimethylarginine (ADMA), an endogenous competitive inhibitor of NOS [16]. On the other hand, cGMP is degraded by phosphodiesterase type 5 (PDE5), which catalyzes the hydrolysis of the cyclic phosphate bond of cGMP to form 5’ GMP [14,15].

A balance of neural stimuli, oxygen tension, potassium channels, and endogenous vasoactive substances preserves pulmonary vascular tone [17]. The pathophysiology of endothelial dysfunction associated with PH is complex and involves multiple contributing factors, including reduced vascular wall shear stress [18], hypoxia [19,20,21], and inflammation [22,23]. Endothelial dysfunction is associated with an imbalance of endogenous vasoactive substances, marked by a decrease in vasodilators such as nitric oxide (NO) and prostaglandins [24,25], and an increase in vasoconstrictors such as endothelin-1 and thromboxane [26,27]. Increments in vascular endothelial growth factor (VEGF) and fibroblast growth factor 2 (FGF2) promote the proliferation of vascular smooth muscle cells [28]. Finally, pulmonary vascular remodeling occurs, leading to increased pulmonary vascular resistance, followed by the development of pulmonary hypertension [29,30].

Human studies have shown that diminished NO levels in the lungs and serum are associated with pulmonary hypertension and increased vascular resistance [31,32]. Similarly, deterioration of eNOS expression has been observed in the lungs [33], leading to pulmonary vasoconstriction and vascular remodeling. Gene transfer of eNOS has been shown to reduce the PH symptoms in experimental animals [34,35]. Conversely, increased NO and eNOS expression levels have also been reported in PH-induced experimental animals [36,37].

NO has been proposed as one of the candidate diagnostic and prognostic biomarkers of PH in humans [17,38]. Given the contradictory data, NO should be validated in large, prospective studies to be clinically accepted as a biomarker in both human and veterinary medicine. In dogs, only one study has revealed that a decrease in NO levels is consistent with the progression of myxomatous mitral valve disease (MMVD) [39]. To date, there has been no study on alterations in the NO/eNOS pathway in dogs with PH. Therefore, this study aims to compare plasma NO and serum eNOS levels between healthy dogs and those with PH, as well as to examine differences in NO/eNOS levels between dogs with PH, with and without ascites. Additionally, the study analyzes the correlations between plasma NO, serum eNOS, and echocardiographic parameters associated with cardiovascular changes.

## 2. Materials and Methods

Animals: The study included a total of twenty-seven dogs, divided into healthy (*n* = 10) and PH (*n* = 17) groups. The PH group dogs were classified into non-ascites (*n* = 6) and ascites (*n* = 11) subgroups. All dogs were client-owned and presented to the Veterinary Teaching Hospital, Khon Kaen University, Thailand, from October 2020 to March 2024. All dog owners provided informed consent prior to the study. The Institutional Animal Care and Use Committee of Khon Kaen University approved the study protocol (IACUC-KKU (C)-40/67).

All dogs received standard health checks and cardiac evaluations, including physical examinations, such as heart rate, respiratory rate, heart murmur sound, point of maximal murmur intensity, and analysis of the presence of jugular vein distention (as determined by visual observation and/or the hepatojugular reflux test), breathing pattern, subcutaneous edema, and ascitic presentation. Systolic and diastolic blood pressure were measured using an oscillometric device (Vet20, SunTech Medical, Morrisville, NC, USA). We conducted thoracic radiography and echocardiographic evaluations in all dogs. In thoracic radiography, we assessed vertebral heart score, heart morphology, pulmonary vessel dimensions, pulmonary lesion patterns, pulmonary edema, pleural effusion, and abdominal effusion. Blood samples were collected from all dogs for routine hematology (complete blood count and blood smear) and blood chemistry analyses, including blood urea nitrogen (BUN), creatinine, alanine transaminase (ALT), total protein, and albumin. Small-breed dogs aged greater than or equal to seven years without cardiac diseases or other systemic diseases were classified in the healthy group. Dogs in the PH group were classified based on the ACVIM consensus statement guidelines [1].

Exclusion criteria were (1) dogs receiving current cardiac or respiratory medications, such as furosemide, pimobendan, angiotensin-converting enzyme inhibitor (ACEi), sildenafil, bronchodilators, vasodilators, or steroids; and (2) dogs with chronic diseases or inflammation, including, liver disease, kidney disease, neoplasia, neurological disorders, or endocrine diseases.

Thoracic imaging: Thoracic radiographs were obtained without anesthesia using a digital radiography machine (VIVIX-S 1717V, Vieworks CO., Anyang, Gyeonggi-do, Korea). The dogs were positioned in lateral recumbency (left or right side down) and in either ventrodorsal (VD) or dorsoventral (DV) orientations. The observations recorded included the vertebral heart score, heart shape, pulmonary infiltration patterns, pulmonary vessel characteristics, and indicators of congestive heart failure, such as pulmonary edema and cardiogenic ascites.

Echocardiography: All echocardiographic measurements were carried out using the Vetus 8 (Shenzhen Mindray Animal Medical Technology Co., Ltd., Shenzhen, China). Each parameter was measured twice to ensure accuracy. Phased array transducers with frequencies of 4–10 MHz, 2–8 MHz, and 2–4 MHz were utilized for dogs weighing less than 5 kg, 5–15 kg, and more than 15 kg, respectively. The echocardiographic parameters are displayed in Table 1.

The left atrium (LA) and main pulmonary artery (MPA) sizes were measured by comparing them to the size of the aorta (Ao) in the B-mode of the right parasternal short-axis view. The LA/Ao and MPA/Ao ratios were then calculated.

Left ventricular measurements were performed at the mid-ventricular level. Both right parasternal short- and long-axis views were utilized with M-mode imaging during both diastole and systole. Measurements included the interventricular septal thickness (IVSd, IVSs), the left ventricular internal diameter (LVIDd, LVIDs), and the left ventricular posterior wall thickness (LVPWd, LVPWs). All left ventricular values were normalized using the allometric equation derived from Cornell’s formula.

The right atrioventricular size was assessed using the right atrial area (RAA) index [9] and the right ventricular end-diastolic area (RVEDA) index [4]. To measure the RAA and RVEDA indices, the internal surfaces of the right atrium and right ventricle were traced during diastole using the left apical four-chamber view, focusing on the right cardiac chamber. The RAA and RVEDA indices were calculated by dividing RAA and RVEDA by body surface area (BSA). BSA was calculated using Formula (1) [40]:

BSA = 0.101 × body weight (kg)^2/3^ (1)

Left ventricular systolic function, including ejection fraction (EF) and fractional shortening (FS), as well as hemodynamic parameters such as heart rate (HR), stroke volume (SV), and cardiac output (CO), were measured using M-mode echocardiography in conjunction with electrocardiography from the right parasternal long axis positioned at the mid-left ventricular level. CO was calculated using the simplified Teicholz formula. Moreover, cardiac index (CI) and stroke volume index (SVI) were calculated using CO/BSA and CI/HR, respectively [41].

Right ventricular systolic function was evaluated using the right ventricular fractional area change (RV FAC) method measured from the left apical four-chamber view and calculated using Equation (2) [5]:

(2) $$\frac{\left(\text{RV diastolic area}-\text{RV systolic area}\right)}{\text{RV diastolic area}}\times 100$$

The right parasternal short-axis view at the pulmonary artery level was used to measure the right pulmonary artery distensibility (RPAD index) by positioning an M-mode cursor at the level of the right pulmonary artery during both the diastolic and systolic phases. The RPAD index was calculated using Equation (3) [7]:

(3) $$\frac{\left(\text{RPADsystole}-\text{RPADdiastole}\right)}{\text{RPADsystole}}\times 100$$

A pulse-wave Doppler probe was pointed at the pulmonic valve area to measure the acceleration-to-ejection time ratio (AT/ET) [8] and the pulmonary valve velocity time integral (PV VTI), both of which were measured in the same echocardiography plane. Continuous-wave Doppler echocardiography was used to measure the peak tricuspid regurgitation velocity (TRVmax) in the left apical four-chamber view, where the tricuspid valve was located. The Bernoulli equation was used to calculate the maximal pressure gradient of tricuspid regurgitation (TRmaxPG) using Equation (4). Pulmonary vascular resistance was calculated using Equation (5) [42]:

PG = 4 × velocity^2^ (m/s) (4)

(5) $$\frac{{TR\;velocity}^{2}(m/s)}{PV\;VTI(cm)}$$

The classification of dogs with PH in this study was based on the ACVIM consensus statement guidelines [2], which categorize the condition into six groups: Group 1 includes pulmonary arterial hypertension (PAH), which is determined by the presence of congenital cardiogenic shunts, PAH occurring in young dogs, or idiopathic PAH; Group 2 refers to PH secondary to left heart disease, identified by the presence of LA enlargement, with LA/Ao ratio greater than 1.6; Group 3 encompasses PH secondary to respiratory disease, hypoxia, or both, and is diagnosed based on a history and clinical signs associated with chronic upper or lower respiratory tract abnormalities, along with evidence of lung parenchyma or airway abnormalities on thoracic radiography, while excluding diffuse pulmonary neoplasia; Group 4 involves PH secondary to pulmonary emboli, which is determined by the presence of pulmonary thrombus or spontaneous echo contrast; Group 5 pertains to PH secondary to parasitic disease, and is identified through the detection of microfilaria on blood smear, Woo’s method, modified Knott test, or a commercial heartworm antigen detection test, with the detection of an equal sign on echocardiography also being considered; and Group 6 refers to PH with multifactorial or unclear mechanisms, where multiple underlying diseases (more than one) associated with PH or the presence of a compressive pulmonary artery mass are discovered.

Measurement of plasma nitric oxide: Plasma was separated from EDTA blood samples by centrifugation and stored at −80 °C. Before measuring NOx levels, all samples were thawed and filtered twice using a 10 kDa ultrafiltration centrifugal device (Nanosep^®^, Pall Laboratory, Port Washington, NY, USA) at 10,000× *g* and 4 °C for 10 min. Plasma nitric oxide levels were indirectly measured to quantify nitrite and nitrate (NOx) levels using a commercial test kit (Nitric oxide assay kit, MyBioSource, San Diego, CA, USA) according to the manufacturer’s instructions. Briefly, nitrate reductase converted plasma nitrate to nitrite, which then reacted with Griess reagent to form a purple compound. NOx levels were then measured at an absorbance of 550 nm using an absorbance microplate reader (BioTek Epoch 2 Microplate Spectrophotometer, Agilent, Santa Clara, CA, USA). All samples were analyzed in duplicate.

Measurement of endothelial nitric oxide synthase: Serum samples were separated from activated clot blood samples by centrifugation and stored at −80 °C. The eNOS levels were measured using enzyme-linked immunosorbent assay (ELISA), using a commercial test kit (Canine eNOS ELISA kit, MyBioSource, CA, USA) according to the manufacturer’s instructions. Briefly, eNOS in the serum samples bound to the canine eNOS antibodies coated on the wells. Then, a biotinylated canine eNOS antibody was added to create a binding site for streptavidin–HRP. After incubation and washing, an acidic stop solution was added to halt the reaction. Finally, serum eNOS levels were measured at an absorbance of 450 nm using an absorbance microplate reader (BioTek Epoch 2 Microplate Spectrophotometer, Agilent, CA, USA). All samples were analyzed in duplicate.

Statistical Analysis: The Shapiro–Wilk test was used to assess the normality of data distribution. For normally distributed variables, analysis of variance (ANOVA) was performed, and results are reported as means ± standard deviations. For non-normally distributed variables, the Mann–Whitney U test was used, with results presented as medians and interquartile ranges [Q1, Q3]. Linear correlation analysis was conducted to examine relationships between variables, using either Pearson’s or Spearman’s correlation coefficients (r) to indicate the strength of the associations. A *p*-value of less than 0.05 was considered statistically significant.

## 3. Results

### 3.1. Animal Population

The study included 27 dogs: 16 males and 11 females. The dogs belonged to 10 breeds: Chihuahua (*n* = 6; 22.22%), Shih Tzu (*n* = 6; 22.22%), Pomeranian (*n* = 4; 14.81%), Miniature Poodle (*n* = 4; 14.81%), Miniature Pinscher (*n* = 2; 7.41%), French Bulldog (*n* = 1; 3.70%), Labrador Retriever (*n* = 1; 3.70%), Papillon (*n* = 1; 3.70%), Pug (*n* = 1; 3.70%), and Yorkshire Terrier (*n* = 1; 3.70%).

The dogs were classified into two groups: healthy dogs (*n* = 10; 37.04%) and dogs with PH (*n* = 17; 62.96%). All dogs with PH had never received any prior treatment. In the PH group, 11 dogs (64.71%) exhibited ascites, while 6 dogs (35.29%) did not. Healthy dogs and dogs with PH were not statistically different in age (8.9 ± 1.97 and 10.72 ± 4.25 years, respectively) and body weight (5.86 ± 3.33 and 6.3 ± 2.28 kg, respectively) (*p* > 0.05).

The analysis included 17 dogs diagnosed with PH. Of these, 13 (76.47%) were classified as having a high probability of PH: 10 dogs (58.82%) had ascites, while 3 dogs (17.65%) did not. The remaining four dogs (23.53%) were classified as having an intermediate probability of PH: one dog (5.88%) had ascites, while three dogs (17.65%) did not. Left-sided heart disease and multifactorial or unclear mechanisms were the predominant underlying causes of PH in this study. Most dogs with PH exhibited clinical signs, with decreased appetite and exercise intolerance. Table 2 presents detailed clinical data for the dogs with PH, both with and without ascites, categorized by PH probability, underlying cause, and clinical signs.

In Group 2, all dogs (*n* = 8) were diagnosed with MMVD, including stage B2 (*n* = 5) and acute stage C (*n* = 3). Group 3 consisted of dogs with respiratory abnormalities, specifically acute pneumonia (*n* = 1) and tracheal collapse accompanied by chronic bronchitis (*n* = 1). In Group 5, one dog was found to have microfilariae on a blood smear and tested positive for heartworm antigen using a commercial detection kit. Group 6 included dogs with comorbid conditions: MMVD stage B2 with chronic respiratory disease (*n* = 3), and cases with an unclear underlying mechanism (*n* = 3).

### 3.2. Hematological and Biochemical Data

The average hematocrit, hemoglobin, and red blood cell counts in dogs with PH (41.33 ± 7.64%, 14.13 ± 2.64 g/dL, and 6.24 ± 1.11 × 10^6^ cells/μL, respectively) were significantly lower than those in healthy dogs (49.1 ± 4.35%, 16.58 ± 1.61 g/dL, and 7.41 ± 0.61 × 10^6^ cells/μL, respectively) (*p* < 0.01). In contrast, the average white blood cell count was significantly higher in dogs with PH (15,386.11 ± 4424.42 cells/μL) compared with healthy dogs (8808.00 ± 2843.67 cells/μL) (*p* < 0.001).

Blood chemistry assessments revealed that the mean BUN was significantly elevated in dogs with PH compared with healthy dogs (31.76 ± 15.23 vs. 17.45 ± 3.49 mg/dL, respectively) (*p* < 0.001). However, total protein and albumin levels in dogs with PH (6.23 ± 1.47 g/dL and 2.43 ± 0.61 g/dL, respectively) were significantly lower than in healthy dogs (7.43 ± 1.02 g/dL and 3.06 ± 0.35 g/dL, respectively) (*p* < 0.05).

### 3.3. Echocardiographic Data

#### 3.3.1. Left Cardiac Parameters

The cardiac chamber size and wall thickness, assessed by echocardiography, are presented in Table 3. The left atrial (LA/Ao) and left ventricular (LVIDdN) sizes were significantly larger in dogs with PH (1.80 ± 0.65 cm and 1.86 ± 0.98 cm, respectively) than in healthy dogs (1.27 ± 0.16 cm and 1.38 ± 0.11 cm, respectively) (*p* < 0.05). Additionally, the left ventricular wall thickness (LVPWdN and LVPWsN) was significantly greater in dogs with PH (0.49 ± 0.14 cm and 0.78 ± 0.20 cm) than in healthy dogs (0.37 ± 0.05 cm and 0.61 ± 0.10 cm) (*p* < 0.01).

The average of left ventricular hemodynamic parameters, including CO, CI, and HR, were significantly higher in dogs with PH (2.89 ± 2.15 L/min, 8.79 ± 7.43 L/min/m^2^ and 150.73 ± 28.63 bpm, respectively) than in healthy dogs (1.76 ± 0.75 L/min, 5.50 ± 1.49 L/min/m^2^ and 127.48 ± 26.25 bpm, respectively) (*p* < 0.05).

#### 3.3.2. Right Cardiac Parameters

Echocardiographic parameters associated with the severity of PH are summarized in Table 4. These parameters include the following:

1. Right Cardiac Chamber Size and MPA: The right cardiac size, including the right atrial size (RAA index) and right ventricular size (RVEDA index), was significantly larger in dogs with PH (12.59 ± 6.21 cm^2^/m^2^ and 13.87 ± 5.50 cm^2^/m^2^, respectively) than in healthy dogs (4.86 ± 0.91 cm^2^/m^2^ and 5.07 ± 2.02 cm^2^/m^2^, respectively) (*p* < 0.001). Additionally, the main pulmonary artery diameter (MPA/Ao) in dogs with PH was greater than in healthy dogs (1.36 ± 0.33 vs. 0.90 ± 0.08) (*p* < 0.001).
2. Estimated Systolic Pulmonary Artery Pressure (PAP) Parameters: The average TRmaxPG was significantly higher in dogs with PH (54.33 ± 18.93 mmHg) compared with healthy dogs (*p* < 0.001). However, tricuspid regurgitation was not observed in healthy dogs, and, therefore, TRmaxPG could not be assessed in this group using echocardiography. In contrast, the AT/ET ratio was significantly lower in dogs with PH (0.24 ± 0.07) than in healthy dogs (0.45 ± 0.03) (*p* < 0.001).
3. Right Ventricular Function Parameters: Right ventricular systolic function, as assessed by the RV FAC, was significantly reduced in dogs with PH (46.86 ± 23.43%) compared with healthy dogs (64.77 ± 12.34%) (*p* < 0.05). Additionally, dogs with PH exhibited significantly lower pulmonary artery compliance, as indicated by the RPAD index, compared with healthy dogs (19.15 ± 10.05% vs. 47.66 ± 7.84%, respectively) (*p* < 0.001). Furthermore, the right ventricular afterload, assessed using PVR, was significantly increased in dogs with PH (2.05 ± 0.76) compared with healthy dogs (*p* < 0.001). In healthy dogs, PVR assessment was not possible due to the absence of measurable tricuspid regurgitation velocity.

#### 3.3.3. Echocardiographic Parameters in Dogs with PH, with and Without Ascites

Echocardiographic evaluations were performed in dogs with PH according to the presence or absence of cardiogenic ascites, as shown in Table 5. There were no significant differences in left ventricular size between dogs with PH with cardiogenic ascites and those with PH without ascites (*p* > 0.05). Additionally, the left ventricular hemodynamic parameters, including CI and SVI, were significantly lower in dogs with PH and cardiogenic ascites (6.68 ± 6.55 L/min and 44.89 ± 39.98 mL/beat/m^2^) compared with those without cardiogenic ascites (12.65 ± 7.96 L/min and 93.81 ± 33.77 mL/beat/m^2^) (*p* < 0.05).

The average right cardiac size, estimated using the RAA and RVEDA indices, was not statistically different (*p* > 0.05). Additionally, the average MPA/Ao ratio in dogs with PH and ascites (1.48 ± 0.35) was significantly greater than in those without ascites (1.17 ± 0.18) (*p* < 0.05).

The mean echocardiographic parameters associated with right cardiac function, including RV systolic function (evaluated using RV FAC) and pulmonary artery compliance (assessed using the RPAD index), were significantly lower in dogs with PH with cardiogenic ascites (31.56 ± 9.91% and 13.78 ± 5.50%, respectively) compared with dogs with PH without cardiogenic ascites (74.92 ± 10.04% and 29.01 ± 9.08%, respectively) (*p* < 0.001). Additionally, the mean pulmonary vascular resistance (PVR) was significantly higher in dogs with PH with cardiogenic ascites (2.37 ± 0.70) compared with those without cardiogenic ascites (1.48 ± 0.52) (*p* < 0.01).

### 3.4. Plasma NO and Serum eNOS Levels

The average plasma NO levels in dogs with PH were significantly higher than in healthy dogs (1.80 ± 1.10 vs. 1.17 ± 0.57 µmol/L, respectively) (*p* < 0.05). Additionally, the average serum eNOS levels were significantly elevated in dogs with PH compared with healthy dogs (382.55 ± 101.42 vs. 265.15 ± 128.65 U/mL, respectively) (*p* = 0.01) (Table 6).

Plasma NO levels were significantly lower in dogs with PH with cardiogenic ascites compared with dogs with PH without cardiogenic ascites (1.37 ± 0.80 vs. 2.59 ± 1.21 μmol/L, respectively; *p* = 0.01). However, no significant difference was observed in serum eNOS levels between dogs with PH with and without ascites (*p* > 0.05) (Table 7).

### 3.5. Correlation Between Plasma NO and Serum eNOS and Echocardiographic Parameters

#### 3.5.1. Correlation Between Plasma NO and Serum eNOS and Left Cardiac Parameters

**Figure 1:**
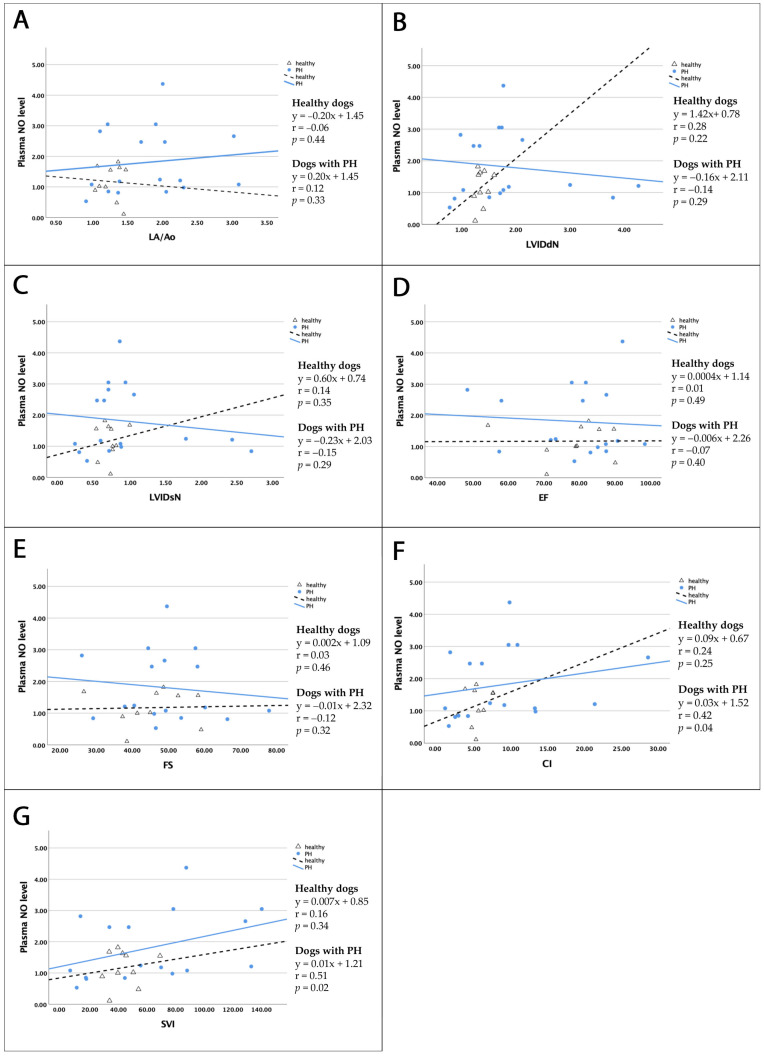
Linear correlations between plasma NO and left-heart echocardiographic variables in healthy dogs (black dotted line) and dogs with PH (blue line). In PH dogs, plasma NO is moderately correlated with CI (**F**) and SVI (**G**) (*p* < 0.05). No significant correlations with plasma NO are observed for LA/Ao (**A**), LVIDdN (**B**), LVIDsN (**C**), EF (**D**), or FS (**E**) in either group. Abbreviations: PH—pulmonary hypertension; NO—nitric oxide; LA/Ao—left atrial to aorta ratio; LVIDdN—normalized left ventricular internal diameter in diastole; LVIDsN—normalized left ventricular internal diameter in systole; EF—ejection fraction; FS—fractional shortening; CI—cardiac index; SVI—stroke volume index.

The results of the correlation analysis between plasma NO and serum eNOS levels and left cardiac echocardiographic parameters in both healthy dogs and dogs with PH are presented in Table 8 and Figure 1. The relationships are indicated by the correlation coefficient “r” values. Plasma NO levels demonstrated moderate positive correlations with hemodynamic parameters, including CI and SVI (r_CI_ = 0.42 and r_SVI_ = 0.51) (*p* < 0.05). However, analysis of the data revealed no significant correlation between plasma NO levels and left cardiac parameters in healthy dogs (*p* > 0.05).

Correlation analysis results between serum eNOS levels and left cardiac parameters are shown in Table 9. No correlation was demonstrated in either healthy dogs or dogs with PH.

#### 3.5.2. Correlation Between Plasma NO and Serum eNOS Levels and Right Cardiac Parameters

**Figure 2:**
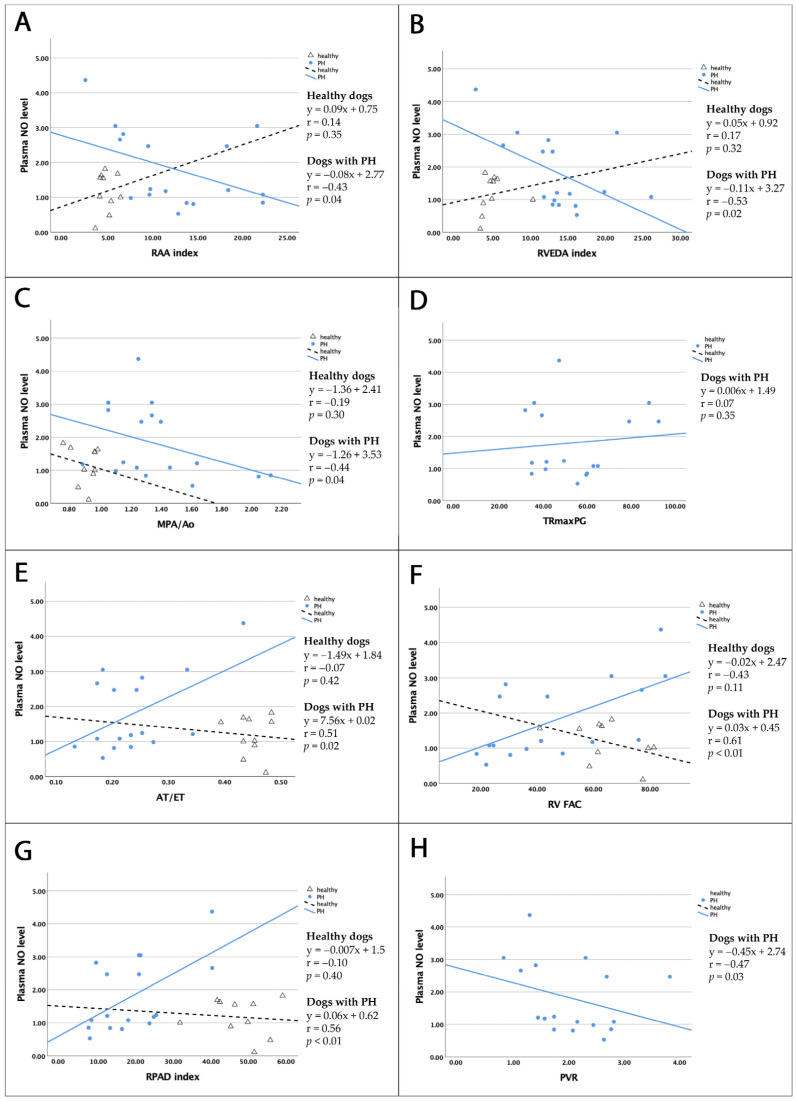
Linear correlations between plasma NO levels and right cardiac echocardiographic parameters in healthy dogs (black dotted line) and dogs with PH (blue line). NO levels in dogs with PH were positively moderately correlated with AT/ET (**E**), RV FAC (**F**), and RPAD index (**G**); negatively weakly correlated with RAA index (**A**), MPA/Ao ratio (**C**), and PVR (**H**); and negatively moderately correlated with RVEDA index (**B**). No correlations were observed between NO levels and TRmaxPG (**D**) (*p* < 0.05). Abbreviations: PH—pulmonary hypertension; NO—nitric oxide; RAA—right atrial area; RVEDA—right ventricular end-diastolic area; MPA/Ao—main pulmonary artery to aorta ratio; AT/ET—acceleration to ejection time ratio; PVR—pulmonary vascular resistance; TRmaxPG—maximal pressure gradient of tricuspid regurgitation; RV FAC—right ventricular fractional area change; RPAD—right pulmonary artery distensibility.

The results of the correlation analysis between plasma NO levels and right cardiac parameters in dogs with PH and healthy dogs are presented in Table 10 and Figure 2, with the relationship denoted by the “r” value. Plasma NO levels showed moderate positive correlations with estimated systolic pulmonary arterial pressure (r_AT/ET_ = 0.51) (*p* = 0.02) and moderate positive correlations with RV contraction and pulmonary distensibility (r_RVFAC_ = 0.61 and r_RPAD_ = 0.56) (*p* < 0.01) in dogs with PH. Additionally, weak negative correlations were observed between plasma NO levels and the RA size (r_RAA_ = −0.43) (*p* = 0.04), while negatively moderate correlations were observed between plasma NO levels and RV size, main pulmonary artery diameter, and pulmonary vascular resistance (r_RVEDA_ = −0.53, r_MPA/Ao_ = −0.44, and r_PVR_ = −0.47, respectively) (*p* < 0.05). No significant correlation was found between plasma NO levels and right echocardiographic parameters in healthy dogs (*p* > 0.05).

Serum eNOS levels were not significantly correlated with echocardiographic parameters reflecting right heart size or function (Table 11).

## 4. Discussion

This study provides a comprehensive evaluation of cardiovascular changes and endothelial function in dogs with pulmonary hypertension (PH), with a particular focus on the role of nitric oxide (NO) and its synthesizing enzyme, endothelial nitric oxide synthase (eNOS). Our findings revealed that the average plasma NO levels in dogs with PH were significantly higher than in healthy controls, and showed a pattern of increasing serum eNOS levels in dogs with PH. Moreover, when comparing the mean NO and eNOS levels in the blood of non-ascites and ascites associated with right-sided congestive heart failure, we found that dogs with PH and ascites had lower NO levels and a pattern of decreasing eNOS levels compared with non-ascites dogs with PH. These results differ from those of previous studies in humans with PH, which generally report lower NO levels and eNOS expression in various samples, including exhaled breath, urine, plasma, and lungs, compared with controls [33,43,44]. However, some studies have reported elevated NO levels or increased eNOS expression in experimental animals with hypoxia-induced PH [36,37,45,46]. A previous study found that hypoxia upregulates eNOS mRNA expression in cultured porcine aortic endothelial cells. This is due to changes in the redox state, which elevate the NAD(P)H/NAD(P) ratio and activate AP-1, leading to increased eNOS expression and NO production [47]. In canine subjects, the severity of hypoxemia is influenced by the extent of elevated systolic PAP; for instance, an average systolic PAP exceeding 40 mmHg has been associated with a reduction in the average partial pressure of oxygen (PaO_2_) to approximately 69 mmHg [48]. However, it is not exactly known whether PH-induced hypoxemia influences NO/eNOS levels in dogs with PH. Increased NO/eNOS levels may reflect a transient compensatory response, with higher levels in dogs with PH but without ascites and lower levels in those with ascites, indicating a stage-dependent decline. Alternatively, the elevated NO levels may be attributed to systemic sampling, which can yield higher values than those obtained from tissue-based measurements, which more accurately reflect local endothelial function. This interpretation aligns with the findings of previous studies that assessed baseline NO levels in both tissue and circulating samples [49,50]. Circulating eNOS levels may also increase due to acute endothelial injury [51] and shedding, rather than active NO synthesis.

In this study, echocardiographic assessment revealed distinct structural and functional alterations characteristic of PH in dogs, notably right atrial and ventricular enlargement, as indicated by increased RAA and RVEDA indices. Our study is consistent with previous veterinary research indicating that progressive RA and RV enlargement can predict right-sided congestive heart failure [4,9]. Moreover, these findings suggest chronic right-sided pressure overload, likely reflecting compensatory remodeling in response to elevated PVR [52]. The increased MPA/Ao ratio further supports the presence of pulmonary arterial distension, with more pronounced dilation in dogs with ascites indicating advanced vascular remodeling and a greater hemodynamic burden [7,53,54]. While indices such as TRmaxPG and the AT/ET ratio effectively confirm elevated pulmonary artery systolic pressure, they do not differentiate between dogs with PH with and without ascites, limiting their utility in staging disease severity. In contrast, reductions in right ventricular fractional area change (FAC) and RPAD index, particularly in dogs with PH and ascites, point to declining right ventricular systolic function and reduced pulmonary arterial compliance, which may indicate progression from a compensatory phase to overt right-sided heart failure. These findings are consistent with previous studies where impaired right ventricular function and decreased compliance were associated with worse clinical outcomes [7,55,56]. Additionally, the significantly elevated PVR observed in dogs with PH and ascites may serve as a non-invasive surrogate marker of advanced disease and right heart failure, especially valuable in clinical settings where invasive measurement is not feasible [42].

This study investigated the relationship between NO levels and various echocardiographic parameters reflecting both functional and structural cardiovascular changes in dogs with PH. Significant associations were observed: (1) a negative correlation with RAA and RVEDA; (2) a negative correlation with MPA/Ao; (3) a positive correlation with RPAD; (4) a positive correlation with AT/ET; (5) a positive correlation with RV FAC; and (6) a negative correlation with PVR. We propose that elevated NO levels may be associated with either compensatory or pathological processes. This interpretation is supported by previous studies [57,58] suggesting that increased eNOS expression may serve as a compensatory mechanism to counteract pulmonary vasoconstriction. Additionally, eNOS downregulation has been reported in patients with progressive congestive heart failure [59]. However, elevated NO/eNOS levels may also represent a transient pathological response to acute endothelial injury caused by pulmonary hypertension [51].

Many previous studies used exogenous NO to counteract pathological cardiovascular changes in PH. For example, researchers demonstrated that exogenous NO inhalation effectively reduced PVR using a dog model of monocrotaline pyrrole-induced chronic pulmonary hypertension [47] and a dog model of cardiomyopathy [60]. Similarly, patients with PH who were treated with NO therapy exhibited lower PVR and improved RV ejection fraction [61]. Additionally, continuous NO inhalation was shown to reduce RV remodeling in a newborn rat model of chronic hypoxia-induced PH [62]. Exogenous NO inhalation also increased NO levels and reduced mean PAP in a dog model of chronic pulmonary hypertension [63]. Furthermore, NO, used as a pulmonary vasodilator, has been shown to enhance pulmonary artery distensibility in patients with PH, as demonstrated through magnetic resonance imaging [64].

Analysis of left cardiac parameters showed that dogs with PH had significantly larger left cardiac dimensions, as assessed using the LA/Ao ratio and LVIDdN, compared with healthy dogs. These findings can be attributed to the fact that the majority of dogs with PH in this study had PH secondary to left-sided heart diseases (*n* = 8/17; 47.06%). However, when comparing the size of the left ventricular chamber in dogs with PH, with and without ascites, dogs with PH and ascites had significantly smaller left ventricular dimensions (LVIDdN) than dogs without ascites. Additionally, evaluation of left ventricular hemodynamic function indicated that dogs with PH and ascites had significantly lower levels of CI and SVI compared with dogs without ascites. This difference may be attributed to the increased PVR observed in dogs with PH with ascites, which was significantly higher than in dogs with PH without ascites. The increase in PVR led to a higher right ventricular afterload, consequently reducing pulmonary circulation and lowering CI and SVI in dogs with PH with CHF. Previous studies similarly indicated a reduction in both left ventricular size [1,7,65,66] and hemodynamic function in dogs with PH [67]. Additionally, plasma NO levels had a moderate positive correlation with left cardiac parameters, including CI and SVI. Under normal conditions, NO acts as a pulmonary vasodilator to improve pulmonary circulation. Consequently, increased NO levels may enhance pulmonary flow, leading to increased circulation in the LA and LV, ultimately resulting in higher CI and SVI.

The hematological values of both the healthy and PH groups were within normal ranges. Although the total WBC of both groups was within normal limits, the total WBC was significantly higher in dogs with PH than in healthy dogs. Similarly, previous studies also showed a small increase in WBCs in dogs with PH [2,66]. It was noted that PH-associated concurrent diseases in the present study (e.g., chronic respiratory diseases and heartworm infections) might affect this finding. The blood biochemistry of both groups was within normal range, except for BUN. A mild increase in the average BUN level (normal range: 8–25 mg/dL) was found in dogs with PH, which was higher than that of healthy dogs. This finding was consistent with that of a previous study, which reported an increase in BUN levels but no increase in creatinine concentrations in both pre-capillary and post-capillary PH [68]. Elevated BUN may result from various factors, mainly dehydration; however, this should be investigated further using urinalysis.

Our study was clinical in nature, involving client-owned dogs, which introduced certain limitations. We could not control the home intake of food, water, or waterborne nitric oxide. However, healthy dogs fasted for at least 6 h to match the PH group conditions. Moreover, client-owned dogs could not undergo invasive procedures, such as cardiac catheterization to directly measure cardiac function or tissue sampling to evaluate eNOS expression in pulmonary vessels. While these methods might provide more accurate results, their use was not feasible in this study. In addition, this study did not use definitive diagnostic tests to confirm the classification of pulmonary hypertension (PH) groups. For example, in Groups 3 and 4, diagnoses were based solely on clinical signs, thoracic radiography, and echocardiography, and more advanced diagnostic modalities such as computed tomography (CT), bronchoscopy, bronchoalveolar lavage, and lung biopsy were not used to confirm PH secondary to respiratory disease or pulmonary thromboembolism. Furthermore, in Group 5, *Angiostrongylus* infection was neither investigated nor confirmed. Another limitation is the relatively small sample size (*n* = 27), particularly within the ascites and non-ascites subgroups. This limited number was primarily due to strict inclusion criteria, which excluded dogs with non-cardiovascular comorbidities commonly observed in older canine populations. Finally, future studies could benefit from incorporating triplicate measurements to further enhance data reliability and reproducibility.

## 5. Conclusions

In this study, cardiovascular changes in dogs with PH were assessed using echocardiography, focusing on right cardiac remodeling, MPA distension, impaired right cardiac function, and estimated sPAP elevation. Notably, dogs with PH that had ascites exhibited significantly worse echocardiographic parameters when compared with those without ascites. Furthermore, dogs with PH and ascites also showed decreased left-sided hemodynamic parameters, such as CI and SVI, suggesting that these dogs were at a more advanced stage of the disease.

Although multiple factors may contribute to elevated NO and eNOS levels in dogs with PH, our findings suggest that these increases may represent a possible compensatory response to cardiovascular changes, particularly in the early stages of the disease. This compensatory mechanism appears to diminish as PH progresses, and other factors—such as measurement methods, hypoxemia, altered oxygen tension, endothelial injury, and systemic inflammation—may further affect NO and eNOS levels. While circulating NO and eNOS are not definitive biomarkers for PH, they can still provide supportive information regarding cardiovascular alterations across both early and advanced disease stages. Further studies utilizing multivariate analysis or logistic regression could help to clarify their associations with echocardiographic parameters and better control for confounding factors.

## Figures and Tables

**Table 1:** Echocardiographic parameters.

Cardiac Evaluation	Echocardiographic Parameters
(1) Left cardiac dimension	LA/Ao LV parameter (IVS, LVID, LVPW) during diastole and systole, normalized using Cornell’s formula
(2) Right cardiac dimension	RAA and RVEDA index MPA/Ao
(3) LV systolic function	FS, EF
(4) Hemodynamic parameters	HR, CO, CI, SV, SVI
(5) Right cardiac function	Estimated sPAP: AT/ET and TRmaxPG RV contraction: RV FAC PA compliance: RPAD index RV afterload: PVR

Abbreviations: LA/Ao—left atrium to aorta ratio; IVS—interventricular septum; LVIDd—left ventricular internal diameter; LVPWd—left ventricular posterior wall diameter; RAA—right atrial area; RVEDA—right ventricular end-diastolic area; MPA/Ao—main pulmonary artery to aorta ratio; LV—left ventricle; FS—fractional shortening; EF—ejection fraction; HR—heart rate; CO—cardiac output; CI—cardiac index; SV—stroke volume; SVI—stroke volume index; sPAP—systolic pulmonary arterial pressure; AT/ET—acceleration to ejection time ratio; TRmaxPG—maximal pressure gradient of tricuspid regurgitation; RV—right ventricle; FAC—fractional area change; PA—pulmonary artery; RPAD—right pulmonary artery distensibility; PVR—pulmonary vascular resistance.

**Table 2:** Clinical data for dogs with PH.

Clinical Data	Dogs with PH Number of Dogs; (%)		
	Ascites PH (*n* = 11; 61.11%)	Non-Ascites PH (*n* = 6; 38.89%)	Total (*n* = 17; 100%)
PH probability			
(1) Intermediate	1; (5.88%)	3; (17.64%)	4; (23.53%)
(2) High	10; (58.82%)	3; (17.64%)	13; (76.47%)
Classification			
(1) Idiopathic	0	0	0
(2) Left-sided heart disease	5; (29.41%)	3; (17.64%)	8; (47.06%)
(3) Chronic respiratory diseases	1; (5.88%)	1; (5.88%)	2; (11.76%)
(4) Pulmonary thromboembolism	0	0	0
(5) Parasitic diseases	1; (5.88%)	0	1; (5.88%)
(6) Multifactorial or unclear mechanisms	4; (23.53%)	2; (11.76%)	6; (35.29%)
Clinical signs			
(1) Decreased appetite	11; (64.71%)	3; (17.64%)	14; (82.35%)
(2) Cough	6; (35.29%)	6; (35.29%)	12; (70.59%)
(3) Dyspnea	8; (47.06%)	3; (17.64%)	11; (64.71%)
(4) Exercise intolerance	11; (64.71%)	6; (35.29%)	17; (100%)
(5) Weight loss	5; (29.41%)	2; (11.76%)	7; (41.18%)
(6) Syncope	3; (17.64%)	1; (5.88%)	4; (23.53%)
(7) Right-sided congestive heart failure	11; (64.71%)	0	11; (64.71%)
(8) Left-sided congestive heart failure	3; (17.64%)	2; (11.76%)	5; (29.41%)

**Table 3:** Assessment of left cardiac size.

Echocardiographic Parameters	Healthy Dogs (*n* = 10)	Dog with PH (*n* = 17)
LA/Ao	1.27 ± 0.16	1.80 ± 0.65 *
IVSdN (cm)	0.39 ± 0.06	0.47 ± 0.13
LVIDdN (cm)	1.38 ± 0.11	1.86 ± 0.98 *
LVPWdN (cm)	0.37 ± 0.05	0.49 ± 0.14 *
IVSsN (cm)	0.60 ± 0.08	0.74 ± 0.30
LVIDsN (cm)	0.72 ± 0.13	0.96 ± 0.70
LVPWsN (cm)	0.61 ± 0.10	0.78 ± 0.20 *
EF (%)	78.46 ± 10.75	79.06 ± 13.46
FS (%)	45.60 ± 9.99	49.44 ± 12.74
HR (bpm)	127.48 ± 26.25	150.73 ± 28.63 *
CO (L/min)	1.76 ± 0.75	2.89 ± 2.15 *
CI (L/min/m^2^)	5.50 ± 1.49	8.79 ± 7.43 *
SV (ml)	13.67 ± 4.66	19.26 ± 12.55
SVI (mL/beat/m^2^)	44.11 ± 11.84	62.16 ± 44.00

Normally distributed data are expressed as mean ± SD. A superscript asterisk (*) indicates significant differences (*p* < 0.05) compared with healthy dogs. Abbreviations: PH—pulmonary hypertension; LA/Ao—left atrial to aorta ratio; IVSdN—normalized interventricular septal diameter in diastole; LVIDdN—normalized left ventricular internal diameter in diastole; LVPWdN—normalized left ventricular posterior wall diameter in diastole; IVSsN—normalized interventricular septal diameter in systole; LVIDsN—normalized left ventricular internal diameter in systole; LVPWsN—normalized left ventricular posterior wall diameter in systole; EF—ejection fraction; FS—fractional shortening; HR—heart rate; CO—cardiac output; CI—cardiac index; SV—stroke volume; SVI—stroke volume index.

**Table 4:** Right cardiac size and function assessment.

Echocardiographic Parameters	Healthy Dogs (*n* = 10)	Dogs with PH (*n* = 17)
RAA index (cm^2^/m^2^)	4.86 ± 0.91	12.59 ± 6.21 *
RVEDA index (cm^2^/m^2^)	5.07 ± 2.02	13.87 ± 5.50 *
MPA/Ao	0.90 ± 0.08	1.36 ± 0.33 *
TRmaxPG (mmHg)	not measurable	54.33 ± 18.93 *
AT/ET	0.45 ± 0.03	0.24 ± 0.07 *
RV FAC (%)	64.77 ± 12.34	46.86 ± 23.43 *
RPAD index (%)	47.66 ± 7.84	19.15 ± 10.05 *
PVR	not measurable	2.05 ± 0.76 *

Normally distributed data are expressed as mean ± SD. A superscript asterisk (*) indicates significant differences (*p* < 0.05) compared with healthy dogs. Abbreviations: PH—pulmonary hypertension; RAA—right atrial area; RVEDA—right ventricular end-diastolic area; MPA/Ao—main pulmonary artery to aorta ratio; TRmaxPG—maximal pressure gradient of tricuspid regurgitation; AT/ET—acceleration to ejection time ratio; RV FAC—right ventricular fractional area change; RPAD—right pulmonary distensibility; PVR—pulmonary vascular resistance.

**Table 5:** Echocardiographic parameters in dogs with PH with and without ascites.

Echocardiographic Parameters		Ascites PH (*n* = 11)	Non-Ascites PH (*n* = 6)
LA and LV size	LA/Ao	1.73 ± 0.68	1.92 ± 0.63
	LVIDdN (cm)	1.76 ± 1.17	2.04 ± 0.50
Left cardiac hemodynamic parameter	CI (L/min)	6.68 ± 6.55	12.65 ± 7.96 *
	SVI (mL/beat/m^2^)	44.89 ± 39.98	93.81 ± 33.77 *
RA and RV size	RAA index (cm^2^/m^2^)	14.21 ± 5.58	9.62 ± 6.70
	RVEDA index (cm^2^/m^2^)	14.68 ± 4.10	12.40 ± 7.70
MPA size	MPA/Ao	1.48 ± 0.35	1.17 ± 0.18 *
Estimated sPAP	TRmaxPG (mmHg)	56.99 ± 18.72	49.44 ± 20.05
	AT/ET	0.22 ± 0.06	0.27 ± 0.10
Right cardiac function	RV FAC (%)	31.56 ± 9.91	74.92 ± 10.04 *
	RPAD index (%)	13.78 ± 5.50	29.01 ± 9.08 *
	PVR	2.37 ± 0.70	1.48 ± 0.52 *

Normally distributed data are expressed as mean ± SD. Non-normally distributed data are expressed as medians and ranges [Q1, Q3]. A superscript asterisk (*) indicates significant differences (*p* < 0.05) compared with dogs with PH and ascites. Abbreviations: PH—pulmonary hypertension; LA/Ao—left atrial to aorta ratio; LVIDdN—normalized left ventricular internal diameter in diastole; CI—cardiac index; SVI—stroke volume index; RAA—right atrial area; RVEDA—right ventricular end-diastolic area; MPA/Ao—main pulmonary artery to aorta ratio; TRmaxPG—maximal pressure gradient of tricuspid regurgitation; AT/ET—acceleration to ejection time ratio; RV FAC—right ventricular fractional area change; RPAD—right pulmonary distensibility; PVR—pulmonary vascular resistance.

**Table 6:** Plasma NO and serum eNOS levels in healthy dogs and dogs with PH.

	Healthy Dogs (*n* = 10)	Dogs with PH (*n* = 17)
Plasma NO (mol/L)	1.17 ± 0.57	1.80 ± 1.10 *
eNOS (U/mL)	265.15 ± 128.65	382.55 ± 101.42 *

Normally distributed data are expressed as mean ± SD. A superscript asterisk (*) indicates significant differences (*p* < 0.05) compared with healthy dogs. Abbreviations: PH—pulmonary hypertension; NO—nitric oxide; eNOS—endothelial nitric oxide synthase.

**Table 7:** Plasma NO and serum eNOS levels in dogs with PH with and without ascites.

	Ascites PH Dogs (*n* = 11)	Non-Ascites PH Dogs (*n* = 6)
Plasma NO (mol/L)	1.37 ± 0.80	2.59 ± 1.21 *
eNOS (U/mL)	366.15 ± 70.83	412.63 ± 145.62

Normally distributed data are expressed as mean ± SD. A superscript asterisk (*) indicates significant differences (*p* < 0.05) compared with dogs with PH and ascites. Abbreviations: PH—pulmonary hypertension; NO—nitric oxide; eNOS—endothelial nitric oxide synthase.

**Table 8:** Correlation between plasma NO levels and left cardiac parameters.

	r in Healthy Dogs	*p*-Value	r in Dogs with PH	*p*-Value
LA/Ao	−0.06	0.44	0.12	0.33
LVIDdN	0.28	0.22	−0.14	0.29
LVIDsN	0.14	0.35	−0.15	0.29
EF	0.01	0.49	−0.07	0.40
FS	0.03	0.46	−0.12	0.32
CI	0.24	0.25	0.42	0.04 **
SVI	0.16	0.34	0.51	0.02 **

Double superscript asterisk (**) indicates significant Spearman’s rho correlation (*p* < 0.05, 1-tailed). Abbreviations: LA/Ao—left atrial to aorta ratio; LVIDdN—normalized left ventricular internal diameter in diastole; LVIDsN—normalized left ventricular internal diameter in systole; EF—ejection fraction; FS—fractional shortening; CI—cardiac index; SVI—stroke volume index; r—correlation coefficient.

**Table 9:** Correlation between serum eNOS levels and left cardiac parameters.

	r in Healthy Dogs	*p*-Value	r in Dogs with PH	*p*-Value
LA/Ao	0.02	0.48	−0.07	0.49
LVIDdN	−0.06	0.44	−0.13	0.28
LVIDsN	0.31	0.19	−0.23	0.28
EF	−0.31	0.19	0.20	0.29
FS	−0.36	0.16	0.33	0.25
CI	−0.27	0.22	−0.03	0.49
SVI	−0.23	0.27	0.23	0.10

Abbreviations: LA/Ao—left atrial to aorta ratio; LVIDdN—normalized left ventricular internal diameter in diastole; LVIDsN—normalized left ventricular internal diameter in systole; EF—ejection fraction; FS—fractional shortening; CI—cardiac index; SVI—stroke volume index; r—correlation coefficient.

**Table 10:** Correlations between plasma NO levels and right cardiac parameters.

	r in Healthy Dogs	*p*-Value	r in Dogs with PH	*p*-Value
RAA index	0.14	0.35	−0.43	0.04 *
RVEDA index	0.17	0.32	−0.53	0.02 *
MPA/Ao	−0.19	0.30	−0.44	0.04 **
TRmaxPG	-	-	0.07	0.35
AT/ET	−0.07	0.42	0.51	0.02 *
RV FAC	−0.43	0.11	0.61	<0.01 *
RPAD index	−0.10	0.40	0.56	<0.01 *
PVR	-	-	−0.47	0.03 **

A superscript asterisk (*) indicates a significant Pearson’s correlation (*p* < 0.05, 1-tailed). Double superscript asterisk (**) indicates a significant Spearman’s rho correlation (*p* < 0.05, 1-tailed). Abbreviations: PH—pulmonary hypertension; RAA—right atrial area; RVEDA—right ventricular end-diastolic area; MPA/Ao—main pulmonary artery to aorta ratio; AT/ET—acceleration to ejection time ratio; TRmaxPG—maximal pressure gradient of tricuspid regurgitation; RV FAC—right ventricular fractional area change; RPAD—right pulmonary artery distensibility; PVR—pulmonary vascular resistance; r—correlation coefficient.

**Table 11:** Correlations between serum eNOS levels and right cardiac parameters.

	r in Healthy Dogs	*p*-Value	r in Dogs with PH	*p*-Value
RAA index	0.41	0.12	0.26	0.22
RVEDA index	0.39	0.14	0.25	0.21
MPA/Ao	−0.51	0.07	0.43	0.06
TRmaxPG	-	-	0.39	0.08
AT/ET	−0.07	0.43	−0.13	0.35
RV FAC	0.56	0.05	0.11	0.26
RPAD index	−0.19	0.30	−0.01	0.47
PVR	-	-	0.18	0.31

Abbreviations: PH—pulmonary hypertension; RAA—right atrial area; RVEDA—right ventricular end-diastolic area; MPA/Ao—main pulmonary artery to aorta ratio; AT/ET—acceleration to ejection time ratio; TRmaxPG—maximal pressure gradient of tricuspid regurgitation; RV FAC—right ventricular fractional area change; RPAD—right pulmonary artery distensibility; PVR—pulmonary vascular resistance; r—correlation coefficient.

## Data Availability

All original study results are included in this article; however, the corresponding author can be contacted for further inquiries.
